# Comparison between regular additional endobiliary radiofrequency ablation and photodynamic therapy in patients with advanced extrahepatic cholangiocarcinoma under systemic chemotherapy

**DOI:** 10.3389/fonc.2023.1227036

**Published:** 2023-08-29

**Authors:** Christian Möhring, Oliver Khan, Taotao Zhou, Farsaneh Sadeghlar, Robert Mahn, Dominik J. Kaczmarek, Leona Dold, Marieta Toma, Milka Marinova, Tim R. Glowka, Hanno Matthaei, Steffen Manekeller, Jörg C. Kalff, Christian P. Strassburg, Tobias J. Weismüller, Maria A. Gonzalez-Carmona

**Affiliations:** ^1^ Department of Medicine I, University Hospital of Bonn, Bonn, Germany; ^2^ Department of Pathology, University Hospital of Bonn, Bonn, Germany; ^3^ Department of Nuclear Medicine, University Hospital Bonn, Bonn, Germany; ^4^ Department of Surgery, University Hospital of Bonn, Bonn, Germany; ^5^ Department of Gastroenterology and Oncology, Vivantes Humboldt Hospital, Berlin, Germany

**Keywords:** biliary tract, radio frequency ablation, extrahepatic cholangiocarcinoma, systemic chemotherapy, photodynamic therapy

## Abstract

**Background and aims:**

Extrahepatic cholangiocarcinoma (eCCA) remains a malignancy with a dismal prognosis. The first-line standard of care includes systemic chemotherapy (SC) and biliary drainage through stenting. Endobiliary ablative techniques, such as photodynamic therapy (ePDT) and radio-frequency ablation (eRFA), have demonstrated feasibility and favorable survival data. This study aimed to compare the oncologic outcome in patients treated with SC and concomitant eRFA or ePDT.

**Method:**

All patients with eCCA were evaluated for study inclusion. Sixty-three patients receiving a combination of SC and at least one endobiliary treatment were retrospectively compared.

**Results:**

Patients were stratified into three groups: SC + ePDT (n = 22), SC + eRFA (n = 28), and SC + ePDT + eRFA (n = 13). The median overall survival (OS) of the whole cohort was 14.2 months with no statistically significant difference between the three therapy groups but a trend to better survival for the group receiving ePDT as well as eRFA, during SC (ePDT + SC, 12.7 months; eRFA + SC, 13.8 months; ePDT + eRFA + SC, 20.2 months; p = 0.112). The multivariate Cox regression and subgroup analysis highlighted the beneficial effect of eRFA on OS. Overall, combined therapy was well tolerated. Only cholangitis occurred more often in the SC + eRFA group.

**Conclusion:**

Additional endobiliary ablative therapies in combination with SC were feasible. Both modalities, eRFA and ePDT, showed a similar benefit in terms of survival. Interestingly, patients receiving both regimes showed the best OS indicating a possible synergism between both ablative therapeutic techniques.

## Introduction

Cholangiocarcinoma is a malignant tumor entity with a rare incidence of about 3/100,000 per year in the Western world but a very aggressive disease progression (1). Whereas the incidence rates of extrahepatic cholangiocarcinoma (eCCA) are stagnating, the incidence rates of intrahepatic cholangiocarcinoma (iCCA) are increasing (2). In recent years, an increase in knowledge resulted in medical advances in diagnosis and therapies for biliary tract cancer (3). Nevertheless, the 5-year survival rate remains at a very disappointing level of 7%–20% (4–6). Radical surgery is still the only curative therapy option and can improve the 5-year survival rate to 50%, but recurrence rates up to 85% after resection are unsatisfactory (7–9).

Recent advances in adjuvant therapies have only minimally improved patient survival and should be further developed (10).

Thus, in the majority of patients, the therapy focused on systemic first-line treatment concepts, especially the use of gemcitabine and cisplatin, which are considered standard therapies (11, 12). The recently published TOPAZ-1 trial demonstrated the beneficial effect of adding durvalumab to gemcitabine and cisplatin (13). Under gemcitabine and cisplatin, patients showed a median overall survival (OS) between 11.1 and 12.2 months (14). The addition of durvalumab to the established regimen improved OS to 12.8 months (13). In case of progress under first-line chemotherapy or intolerance, mFOLFOX is available as a second-line therapy (15). The effectiveness was confirmed in a recently published phase III trial with a median OS of 6.2 months [95% confidence interval (CI) = 0.54–0.76] (16). Most importantly, if molecular stratification of the tumor is possible, then immunological treatment or molecularly targeted options should be examined, such as fibroblast growth factor receptor 2 (FGFR2) fusion eligible for treatment with pemigatinib, which has been recently approved, isocitrate dehydrogenase-1 (IDH-1) mutation eligible for treatment with ivosidenib, or microsatellite instability eligible for treatment with pembrolizumab (17–19). With the implementation of next-generation sequencing technology, further targeted therapies will gradually be established for the treatment of cholangiocarcinoma (20).

Because extrahepatic biliary tract cancer is usually associated with bile duct obstruction, supportive endoscopy with biliary stent implantation is essential (21). In combination with this intervention, endobiliary photodynamic therapy (ePDT) and endobiliary radio-frequency ablation (eRFA) are available as local ablative procedures (22). Additional ePDT or eRFA treatment combined with biliary stent placement seemed to be beneficial in the first-line treatment of these patients, because, in several retrospective trials, they have shown improved outcomes concerning OS and stent patency compared with biliary stenting alone (23–29).

These methods can also be used in combination with standard first-line chemotherapy treatment. We and others already reported that the combination of chemotherapy and ePDT is feasible and superior to ePDT or systemic chemotherapy (SC) alone (30–32). Likewise, there have been some retrospective and even prospective studies showing a beneficial role for concomitant eRFA to SC in terms of survival for the treatment of advanced eCCA (33–35). Recently, a systemic review and meta-analysis confirmed these findings and showed an improved survival for ePDT and SC (36).

However, comparative data on ePDT *vs*. eRFA are rare, and data on both therapies with simultaneous SC are missing. Strand et al. conducted a retrospective study with 48 patients in two therapy arms (eRFA, 16 patients; ePDT, 32 patients). They reported no significant difference in OS between these two interventions [median survival of 9.6 (eRFA) versus 7.5 (ePDT) months with p = 0.799, log-rank]. However, in this trial, only 56.2% of patients received chemotherapy (37).

Thus, the aim of the present study is to analyze real-life data of patients with advanced eCCA and compare the therapy regimes (eRFA + SC *vs*. ePDT + SC *vs*. eRFA + ePDT + SC) in terms of OS, progression-free survival (PFS), and tolerability.

## Materials and methods

### Patient population

A total of 419 patients with histologically confirmed cholangiocarcinoma were evaluated for inclusion. Patients were included when they fulfilled the following inclusion criteria: (1) systemic first-line SC with gemcitabine ± platinum derivate and (2) patients who required biliary stenting and agreed to additional endobiliary treatment with ePDT and/or eRFA. Patients were excluded when they received ePDT, eRFA, or SC as monotherapy or received surgery alone or none tumor specific therapy. Patients were also excluded when they were treated with SC in an adjuvant intention or received another SC than gemcitabine ± platinum derivate. Patients were consecutively enrolled in the study.

The main reasons for an unresectable stage of disease were an advanced stage of disease (vascular invasion corresponding T4 stage of TNM Classification of Malignant Tumors (TNM) classification, distant metastasis corresponding N2, and/or M1 stages of TNM classification) or low performance status due to various comorbidities.

If the patient’s condition, renal and hepatic status, permitted, SC was applied first-line treatment. An interdisciplinary team of experts considering the possible side effects and in agreement with the individual patient wishes generated the individualized treatment regimen for each patient.

Patients were classified depending on the treatment regimen: combination of eRFA + SC (n = 28), combination of ePDT + SC (n = 22), or combination of eRFA + ePDT + SC (n =13).

### Data collection and study design

This is a single-institution retrospective three-armed analysis of patients with non-resectable extrahepatic biliary tract cancer who were treated with either standard SC combined with ePDT or eRFA or both locoregional therapy modalities between January 2006 and December 2021. Baseline parameters were recorded prior to therapy. Patients were followed until death; they were lost to follow-up or the end of observation period in October 2022. Patients who were lost to follow-up were censored at the date of last visit. Computer tomography and/or magnetic resonance imaging were used to regularly assess tumor response every 8–12 weeks. Primary endpoints of the analysis were OS and PFS. OS was defined as the time elapsed between administration of the first-line tumor-specific therapy and death or lost to follow-up. The median PFS was defined as the time elapsed between the start of first-line tumor-specific therapy and the onset of progressive disease (PD), lost to follow-up, or death. Secondary endpoints were ORR (objective response rate) and DCR (disease control rate), as well as toxicity assessment. Tumor response was classified as complete or partial remission (CR and PR, respectively), stable disease (SD), or PD based on the radiological imaging evaluation, according to the assessment guidelines in solid tumors (Response Evaluation Criteria In Solid Tumors (RECIST), v1.1). Toxicity was measured using the Common Terminology Criteria for Adverse Events (version v5.0). The responsible ethics committee of the Medical Faculty of the University Bonn had given its approval and had no objections to the study (No. 341/17).

### Therapeutic procedures

#### Biliary stenting

The main indication for endobiliary stenting was jaundice, pruritus, or cholangitis. In addition, a recently diagnosed biliary dilatation, even when asymptomatic, was an indication for endoscopic retrograde cholangiography (ERC)–guided forceps biopsy from the stricture to secure diagnosis of eCCA. In these cases, stents were implanted after biopsy to secure bile flow and to prevent from post-ERC cholangitis. Furthermore, we implanted stents routinely following ePDT or eRFA to prevent from post-ERC cholangitis.

For treatment and prevention of cholestasis, all patients received biliary stents using ERC. Plastic stents (7Fr or 10Fr double-pigtail stents, ENDO-FLEX, Voerde, Germany) were replaced every 3 months to avoid infection and obstruction or earlier if signs of stent dysfunction were present. Metal stents (covered or uncovered 10-mm Wallstent™, Boston Scientific, Marlborough, MA, USA) were used in cases of recurrent early failure of plastic stents or if patient’s performance status did not enable scheduled stent replacements. If ERC biliary stenting was not possible (in three patients: two patients with eRFA + SC and one patient with ePDT + SC), then cholestasis was treated by percutaneous transhepatic cholangiodrainage.

#### Systemic chemotherapy

Most patients in this study received combined first-line SC with gemcitabine (1,000 mg/m^2^) and cisplatin (25 mg/m^2^) on days 1 and 8 every 3 weeks as detailed by the ABC-02 trial11. In patients with impaired renal function or decreasing renal function after cisplatin therapy, oxaliplatin (80 mg/m^2^) was used instead of cisplatin. Patients with poor performance were treated with gemcitabine monotherapy.

#### Endobiliary photodynamic therapy

ERC was used to administer locally photodynamic treatment. Previously implanted biliary stents and intraductal debris were removed before performing ePDT. The chlorine derivate Foscan^®^ (0.04 mg/kg) or the porphyrin derivatives Photosan^®^ (1.5–2.5 mg/kg) and, respectively, Photofrin^®^ (2 mg/kg) were applied. According to the manufacturer’s instructions, the photosensitizing agent was administered intravenously 48 h (Photosan^®^ or Photofrin^®^) or 24 h (Foscan^®^) before treatment. Laser light at an agent-specific wavelength of maximum absorption of the agent was delivered intraluminally with a cylindrical diffuser catheter (30 mm, RD 10‐245, by MEDlight SA, Ecumbens, Switzerland) to stimulate the photosensitizing agent. The activated photosensitizers triggered phototoxic reactions in the treated area, which specifically damaged the malignant cells. The ideal position of the light diffusor in the core of the malignant stricture was verified by fluoroscopy. Plastic stents were placed over the malignant stricture after ePDT was finished. To avoid toxicities, patients were told to protect their skin and eyes from sunlight. The treatment was regularly repeated every 3–4 months, if possible.

#### Endobiliary radio-frequency ablation

This procedure was also conducted via ERC and prepared just like in ePDT. Thereafter, an 8-Fr RFA probe (Habib EndoHPB Bipolar Radiofrequency Catheter, Boston Scientific, Marlborough, MA, USA) was inserted into the narrowed duct using a guide wire. Next, a 90-s cylindrical ablation was performed then across a 25-mm length (VIO 200, Soft Coag mode, effect 8, 10 W, ERBE, Tübingen, Germany). The electrodes were heated by high-frequency electric current to such an extent that the affected cells were precisely damaged. Before further replacement, the electrode was cooled for 60 s. For longer strictures, progressive ablation from proximal to distal was accomplished. Plastic stents were implanted after the eRFA to secure biliary drainage and decompression of the stricture. The treatment was performed every 3–4 months if possible.

### Endoscopic therapy decision-making

The assignment to either ePDT or eRFA was mainly based on endoscopic criteria and potential side effects. eRFA was only feasible if an 8-Fr catheter could traverse the biliary stricture; otherwise, ePDT was used because of its thinner probe. Switching the type of endobiliary therapy was primarily done because of toxicity concerns, patient preference, or the inability to further treat the stricture with RFA. In some cases, lack of improvement in the stricture, as analyzed by ERC, prompted a change in technique.

Endobiliary ablative treatments were repeated as long as they were technically feasible, improved the stricture, and well tolerated by the patient.

### Statistical analysis

Testing for normal distribution of continuous variables was performed with the Shapiro–Wilk test. Continuous variables are expressed as medians and first and third quartiles. In case of normal distribution, differences between three groups were calculated with an ANOVA and in case of non-normal distribution with the Kruskal–Wallis test. Differences between two groups were assessed using the non-parametric Mann–Whitney test. Categorical variables are shown as absolute frequencies and percentages. The Pearson’s Chi-squared test or the Fisher’s exact test was used to compare them. Estimated survivals were compared by log-rank test and displayed in Kaplan–Meier curves and further presented as medians with 95% CI. Univariate and multivariate analyses were performed using Cox regression forward conditional models. If parameters showed p-values ≤0.05 in univariate analysis, then they were included in multivariate analysis. Two-tailored p-values ≤0.05 were considered statistically significant. SPSS version 22 (IBM Corporation, Armonk, NY, USA) was used for statistical analysis.

## Results

### Baseline characteristics

**Figure 1:**
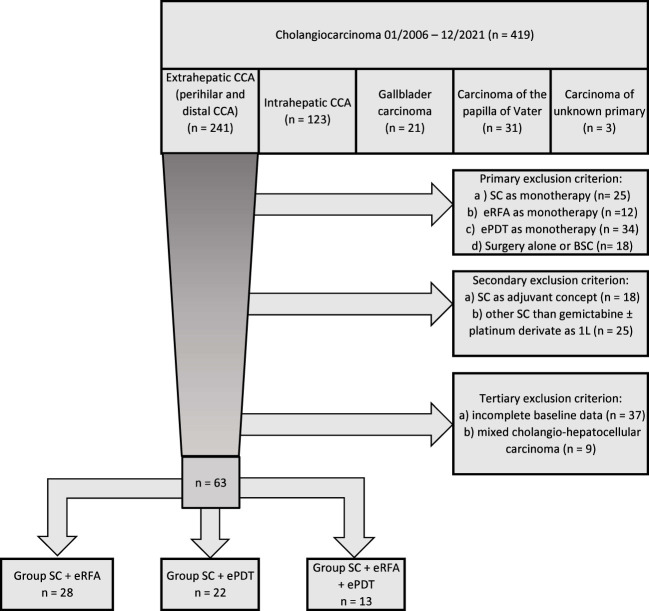
Flow chart.

In total, 419 patients were evaluated for study inclusion (**Figure 1**). Considering the inclusion and exclusion criteria, 63 patients were included in the final analysis. **Table 1** shows the baseline characteristics of all patients. The majority of patients were men (55.6%), and the whole cohort had a median age of 69.0 years with no significant difference between the groups (p = 0.163). The most common local tumor stage present was Bismuth II or IV carcinoma (69.8%). In 38.1% of the patients, a metastatic disease was present at the time of diagnosis. No significant differences were presented among the three groups regarding tumor stage parameters except for tumor grading (p = 0.638 for tumor localization, p = 0.404 for M-stage, p = 0.052 for N-stage, and p < 0.001 for tumor grading). A total of 82.5% of patients had a good or slightly reduced performance status (ECOG 0-1), and this was also similar among the three groups (p = 0.153). No statistically significant differences concerning other relevant oncologic baseline parameters between the three study groups were detected.

**Table 1:** Baseline characteristics.

Parameters			SC + ePDT (N = 22)		SC + eRFA (N = 28)		SC + ePDT + eRFA (N = 13)		P-value		
			N	%	N	%	N	%			
**Sex**									0.678		
Male			12	54.5%	17	60.7%	6	46.2%			
Female			10	45.5%	11	39.3%	7	53.8%			
**Age grouped**									0.115		
< 70			15	68.2%	13	46.4%	10	76.9%			
> 70			7	31.8%	15	53.6%	3	23.1%			
**Risk factors**											
Liver cirrhosis			1	4.5%	2	7.1%	1	7.7%	0.910		
PSC			1	4.5%	2	7.1%	2	15.4%	0.507		
Cholecystolithiasis			9	40.9%	10	35.7%	4	30.8%	0.829		
Diabetes mellitus			2	9.1%	7	25.0%	5	38.5%	0.116		
Smoker or smoking history			5	22.7%	10	35.7%	5	38.5%	0.522		
Hyperlipidemia			5	22.7%	4	14.3%	1	7.7%	0.478		
NAFLD			1	4.5%	2	7.1%	3	23.1%	0.166		
Hepatitis B infection			2	9.1%	1	3.6%	1	7.7%	0.711		
Hepatitis C infection			0	0.0%	1	3.6%	0	0.0%	0.530		
**Grading**									**0.001**		
G1			1	4.5%	4	14.3%	5	38.5%			
G2			3	13.6%	10	35.7%	7	53.8%			
G3			6	27.3%	9	32.1%	0	0.0%			
G4			0	0.0%	1	3.6%	0	0.0%			
Grading unknown			12	54.5%	4	14.3%	1	7.7%			
**Tumor localization**									0.638		
Bismuth I–II			2	9.1%	5	17.9%	2	15.4%			
Bismuth III–IV			16	72.7%	18	64.3%	10	76.9%			
Distal CCA			4	18.2%	3	10.7%	1	7.7%			
**M-Stage**									0.404		
M0			13	59.1%	17	60.7%	4	30.8%			
M1			8	36.4%	9	32.1%	7	53.8%			
MX			1	4.5%	2	7.1%	2	15.4%			
**Metastasis localization**									0.529		
Liver			1	4.5%	2	7.1%	3	23.1%			
Peritoneum			3	13.6%	4	14.3%	3	23.1%			
Other			4	18.2%	1	3.6%	2	15.4%			
**> 1 Metastasis localization**			1	4.5%	2	7.1%	0	0.0%			
**ECOG**									0.153		
0			6	27.3%	13	46.4%	9	69.2%			
1			12	54.5%	10	35.7%	2	15.4%			
2			4	18.2%	5	17.9%	2	15.4%			
	Median	Q1	Q3	Median	Q1	Q3	Median	Q1	Q3	P-value
Age at initial diagnosis	66.5	56.5	71.5	74.0	56.8	79.0	69.0	56.5	70.0	0.163
MELD	5.0	1.8	12.5	9.1	2.7	12.8	3.5	1.4	11.0	0.598
Biochemical conditions at therapy start										
CA 19-9	561.3	136.7	1788.0	203.4	42.4	735.2	210.9	21.0	2340.4	0.367
CEA	9.1	2.1	36.4	2.5	1.3	6.0	2.7	2.1	4.5	0.343
Bilirubin	2.1	1.0	8.8	2.2	0.6	4.9	1.3	0.7	7.6	0.316
gGT	811.0	309.0	1284.3	681.5	298.0	1029.5	1217.0	473.0	1348.0	0.389

CA19-9, carbohydrate antigen 19-9; CCA, cholangiocarcinoma; CEA, carcinoembryonic antigen; ECOG, Eastern Cooperative Oncology Group performance status; ECOG, Eastern Cooperative Oncology; gGT, Gamma-glutamyltransferase; MELD, model for end-stage liver disease; NAFLD, Nonalcoholic fatty liver disease; PSC, primary sclerosing cholangitis.Statistically significant P-Values are displayed in bold values.

### Therapy characteristics

Therapy characteristics are summarized in **Table 2**. All patients were treated with SC as the standard first-line treatment. The majority of patients (71.4%) received SC with gemcitabine and cisplatin as the standard first-line treatment, 1.6% received oxaliplatin instead of cisplatin, and 27% received gemcitabine as monotherapy (p = 0.617). The cohort had no significant difference in number of applied cycles (6 *vs*. 7.5 *vs*. 6 cycles, p = 0.482) during the first-line treatment. Furthermore, 21 patients (33.3%) received more than one line of SC. All patients received additional endobiliary therapy. As the group allocation of patients suggests, 35 patients (55.6%) received at least one session of ePDT and 41 patients (65.1%) at least one session of eRFA. Patients received a median of two ePDT sessions with no difference between the ePDT + SC group and the ePDT + eRFA + SC group. eRFA was applied with a median number of two treatment sessions, and the eRFA + SC group received significant more therapy sessions than the ePDT + eRFA + SC group (p = 0.020). The whole cohort received a median of eight ERC interventions (p = 0.203). Nineteen patients (30.2%) underwent a metal stent placement during therapy (p = 0.369).

**Table 2:** Therapy and therapy response.

Parameters			SC + ePDT (N = 22)		SC + eRFA (N = 28)		SC + ePDT + eRFA (N = 13)		P-value		
			N	%	N	%	N	%			
**SC**			22	100.0%	28	100.0%	13	100.0%	n.a.		
**First-line protocol**									0.617		
GemCis			14	63.6%	21	75.0%	10	76.9%			
GemOx			0	0.0%	1	3.6%	0	0.0%			
GemMono			8	36.4%	6	21.4%	3	23.1%			
Other			0	0.0%	0	0.0%	0	0.0%			
**Dose reduction over time**			11	50.0%	13	46.4%	9	69.2%	0.669		
**Second line of SC**			5	22.7%	8	28.6%	8	61.5%	**0.048**		
**Third line of SC**			2	9.1%	2	7.1%	2	15.4%	0.702		
**ePDT**			22	100.0%	0	0.0%	13	100.0%	n.a.		
**eRFA**			0	0.0%	28	100.0%	13	100.0%	n.a.		
**Surgery**			16	72.7%	17	60.7%	10	76.9%	0.499		
**Type of surgery**									0.854		
No surgery			5	22.7%	9	32.1%	5	38.5%			
Exploration only, without resection			13	59.1%	11	39.3%	5	38.5%			
Resection with curative intention			2	9.1%	4	14.3%	1	7.7%			
Palliative surgery			2	9.1%	4	14.3%	2	15.4%			
**R status after resection**									0.610		
R0			0	0.0%	1	3.6%	0	0.0%			
R1			0	0.0%	2	7.1%	1	7.7%			
Unknown			2	9.1%	1	3.6%	0	0.0%			
**Metal stent**			9	40.9%	6	21.4%	4	30.8%	0.369		
Best Response											
CR			0	0.0%	0	0.0%	0	0.0%	n.a.		
PR			1	4.5%	6	21.4%	1	7.7%	0.171		
SD			11	50.0%	15	53.6%	10	76.9%	0.262		
PD			10	45.5%	7	25.0%	3	23.1%	0.229		
**ORR**			1	4.5%	6	21.4%	1	7.7%	0.171		
**DCR**			12	54.5%	21	75.0%	10	76.9%	0.229		
	Median	Q1	Q3	Median	Q1	Q3	Median	Q1	Q3	P-value	
Number of cycles first line	6.0	2.0	8.0	7.5	1.0	11.8	6.0	3.5	12.5	0.482
Number of cycles second line	5.0	2.0	8.5	3.0	2.0	10.0	3.0	2.0	10.0	0.901
Number of ePDT	2.0	1.0	3.3				3.0	1.5	4.5	0.230
Number of eRFA				3.0	1.0	4.0	1.0	1.0	2.0	**0.020**
Number of ERCP	5.0	12.3	8.0	5.0	10.0	10.0	7.0	16.0	5.0	0.203

CR, complete response; ePDT, endobiliary photodynamic therapy; eRFA, endobiliary radio-frequency ablation; DCR, disease control rate; ORR, objective response rate; PD, progressive disease; PR, partial response; SC, systemic chemotherapy; SD, stable disease.n.a., not applicable.Statistically significant P-Values are displayed in bold values.

### Efficacy

**Figure 2:**
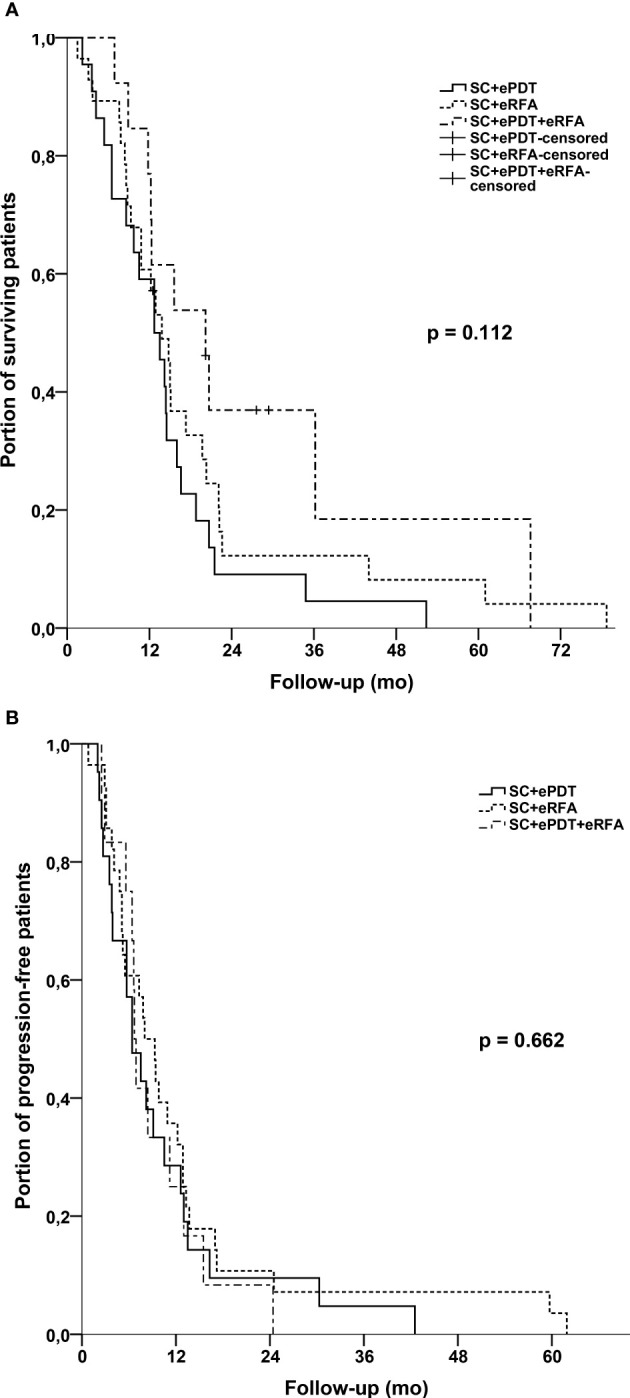
**(A)** Kaplan–Meier estimates with log-rank p for overall survival: SC + eRFA *vs*. SC + ePDT *vs*. SC + eRFA + ePDT. **(B)** Kaplan–Meier estimates with log-rank p for progression-free survival: SC + eRFA *vs*. SC + ePDT *vs*. SC + eRFA + ePDT.

The median follow-up was 13.5 months. Median OS of the whole cohort was 14.2 months (95% CI, 12.0–16.4) with no statistically significant difference between the three therapy groups [ePDT + SC, 12.7 months (95% CI, 8.4–17.0); eRFA + SC, 13.8 months (95% CI, 9.8–17.8); ePDT + eRFA + SC, 20.2 months (95% CI, 11.0–29.4); p = 0.112] (**Figure 2A**). The landmark analysis for 3, 6, 12, and 18 months provided the following survival rates: ePDT + SC: 82%, 68%, 32%, and 14%; eRFA + SC: 89%, 71%, 46%, and 25%; ePDT + eRFA + SC: 100%, 85%, 62%, and 37%. Moreover, the PFS was 7.5 months (95% CI, 5.8–9.2) and likewise showed no difference between the therapy groups [ePDT + SC, 6.4 months (95% CI, 3.7–9.0); eRFA + SC, 8.0 months (95% CI, 5.3–10.7); ePDT + eRFA + SC, 6.7 months (95% CI, 6.2–7.2); p = 0.662] (**Figure 2B**). The ORR was 4.5% for the ePDT + SC group, 21.4% for the eRFA + SC group, and 7.7% for the ePDT + eRFA + SC group (p = 0.171), whereas the DCR was 54.5% for the ePDT + SC group, 75.0% for the eRFA + SC group, and 76.9% for the ePDT + eRFA + SC group (p = 0.229) (**Table 2**).

### Prognostic factors and subgroup analysis

Univariate Cox regression analysis revealed a statistically significant influence on survival for the therapy with eRFA (HR, 0.52; 95% CI, 0.29–0.91), application of more than one SC line (HR, 0.52; 95% CI, 0.29–0.94), surgery with resection in the previous therapies (HR, 0.53; 95% CI, 0.29–0.99), surgery without resection in the previous therapies (HR, 0.35; 95% CI, 0.13–0.97), metal stent placement (HR, 0.42; 95% CI, 0.22–0.78), tumor grading (HR, 2.26; 95%: 1.14–4.49), and DCR (HR, 0.53; 95% CI, 0.30–0.93) (**Table 3**). In the multivariate Cox regression analysis, eRFA (HR, 0.42; 95% CI, 0.18–0.95) and metal stent placement (HR, 0.41; 95% CI, 0.17–0.95) remained the independent predictors of survival.

**Table 3:** Univariate and multivariate analysis.

Parameters	Univariate				Multivariate (forward conditional)			
	Sig.	Exp (B)	95% CI for Exp (B)		Sig.	Exp (B)	95% CI for Exp (B)	
**Male gender**	0.852	0.951	0.564	1.606				
**Age**	0.392	0.990	0.967	1.013				
**Bismuth I–II**	0.881	Ref.						
**Bismuth III–IV**	0.624	0.823	0.378	1.791				
**Distal CCA**	0.923	1.051	0.385	2.867				
**Localization unknown**	0.634	0.682	0.141	3.291				
**M0 *vs*. M1**	0.523	0.968	0.874	1.071				
**Hepatic metastatic disease**	0.955	1.027	0.408	2.587				
**ECOG 0 *vs*. >0**	0.183	1.438	0.843	2.452				
**Total bilirubin (<1.5 *vs*. >1.5)**	0.821	1.063	0.627	1.801				
**CA19-9 (<250 *vs*. >250)**	0.268	0.737	0.430	1.264				
**MELD-Score (<6.5 *vs*. >6.5)**	0.766	1.084	0.638	1.841				
**eRFA**	**0.021**	0.515	0.293	0.905	**0.037**	0.416	0.183	0.947
**ePDT**	0.994	1.002	0.593	1.695				
**1 line *vs*. >1 line SC**	**0.030**	0.523	0.291	0.939				
**No surgery**	0.108	Ref.						
**Surgery with tumor resection**	**0.046**	0.534	0.289	0.989				
**Surgery without tumor resection**	**0.043**	0.348	0.125	0.966				
**Palliative surgery**	0.660	0.822	0.343	1.971				
**R0 *vs*. R1**	0.204	0.899	0.764	1.059				
**Metal stent implantation**	**0.006**	0.416	0.223	0.777	**0.037**	0.406	0.174	0.948
**ORR**	0.800	0.907	0.426	1.932				
**DCR**	**0.027**	0.526	0.297	0.930				
**First-line dose reduction over time**	0.509	0.823	0.461	1.468				
**Tumor grading 1/2 *vs*. 3/4**	**0.020**	2.260	1.138	4.489				

CA19-9, carbohydrate antigen 19-9; CCA, cholangiocarcinoma; DCR, disease control rate; ECOG, Eastern Cooperative Oncology Group performance status; ePDT, endobiliary photodynamic therapy; eRFA, endobiliary radio-frequency ablation; MELD, model for end-stage liver disease; ORR, objective response rate; SC, systemic chemotherapy.Statistically significant P-Values are displayed in bold values.

The M-stage had no statistical influence on the total cohort’s OS with an OS of 14.8 months (95% CI, 10.7–18.9) for locally advanced eCCA, 12.2 months (95% CI, 6.3–18.1) for the cases with metastatic disease, and 13.8 (95% CI, 9.5–18.1) for the MX stage (p = 0.205) (**Supplemental Data 1**). The same effect was observed when stratifying the cohort according to M-stage and comparing two therapy subgroups: patients starting endobiliary ablation with ePDT *vs*. patients starting with eRFA. Patients with locally advanced disease and M0 stage showed a trend toward better median OS when first treated with eRFA (19.7 months; 95% CI, 13.9–25.5) compared with ePDT (12.7 months; 95% CI, 8.9–16.5) but without statistical significance (p = 0.068) (**Supplemental Data 2A**). This trend disappears in metastatic disease (M1) with an OS of 12.2 months (95% CI, 4.9–19.5) for starting eRFA *vs*. 12.7 months (95% CI, 6.7–18.7) for starting ePDT (p = 0.446) (**Supplemental Data 2B**).

**Figure 3:**
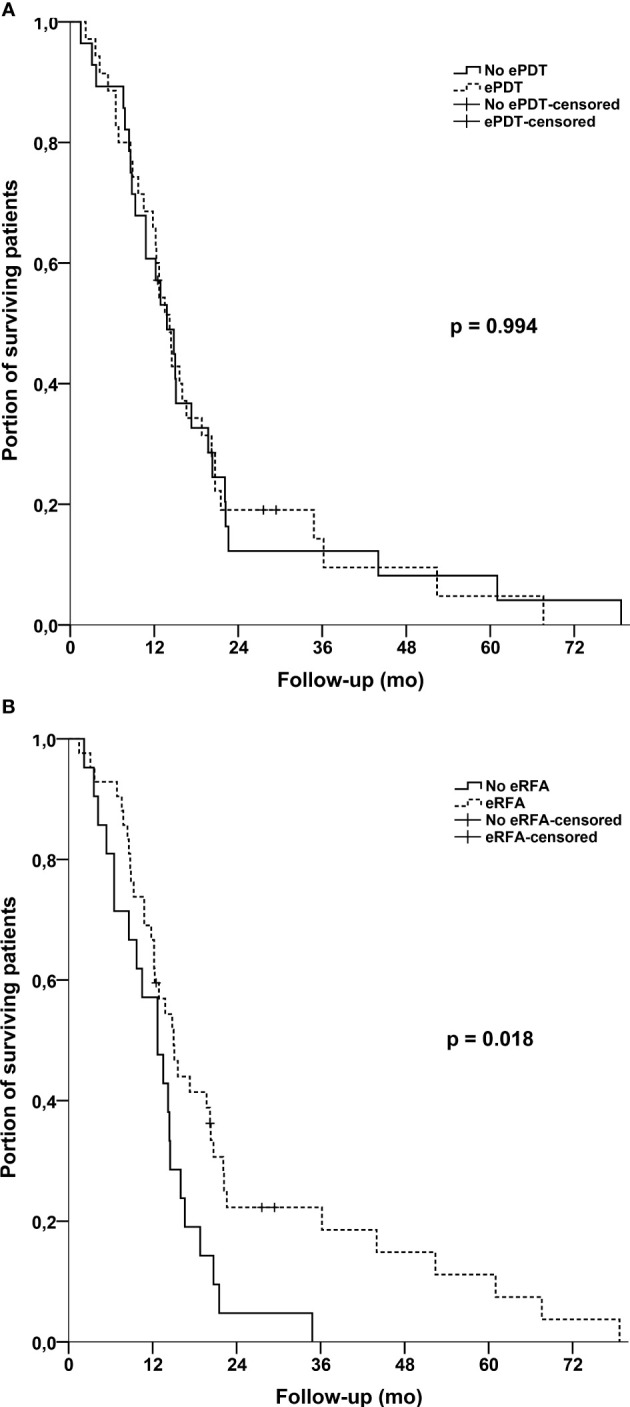
**(A)** Kaplan–Meier estimates with log-rank p for overall survival: No ePDT *vs*. ePDT for the whole cohort. **(B)** Kaplan–Meier estimates with log-rank p for overall survival: No eRFA *vs*. eRFA for the whole cohort.

**Figure 4:**
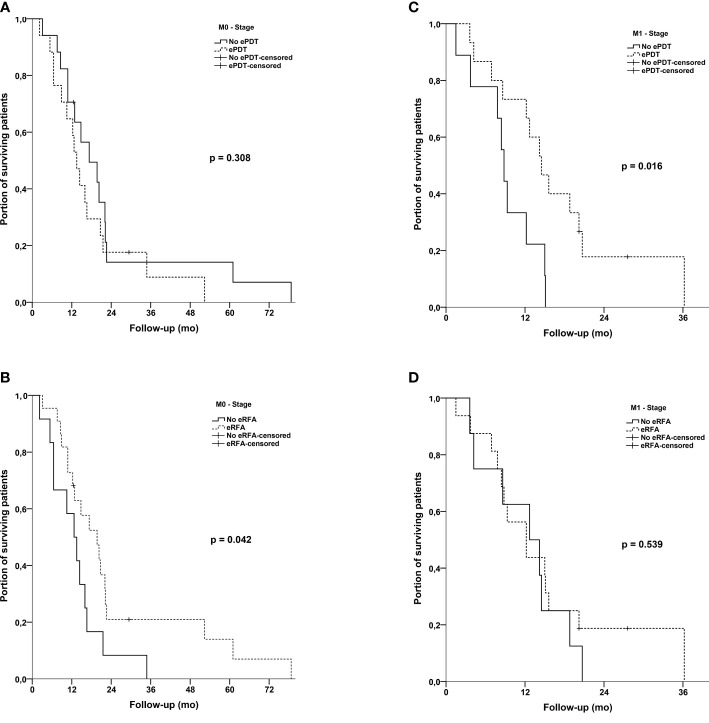
**(A)** Kaplan–Meier estimates with log-rank p for overall survival: No ePDT *vs*. ePDT in M0 stage patients. **(B)** Kaplan–Meier estimates with log-rank p for overall survival: No eRFA *vs*. eRFA in M0 stage patients. **(C)** Kaplan–Meier estimates with log-rank p for overall survival: No ePDT *vs*. ePDT in M1 stage patients. **(D)** Kaplan–Meier estimates with log-rank p for overall survival: No eRFA *vs*. eRFA in M1 stage patients.

In further subgroup analyses, we separately compared patients who received either ePDT or eRFA with patients who did not receive the particular treatment (**Figures 3A, B**). For ePDT, there was no difference in median OS [ePDT: 14.2 months (95% CI, 12.1–16.3) *vs*. no ePDT: 13.8 months (95% CI, 9.8–17.8); p = 0.994]. Interestingly, the log-rank test showed a statistically significant difference for eRFA [eRFA: 15.0 months (95% CI, 11.8–18.2) *vs*. no eRFA: 12.7 months (95% CI, 8.2–17.2); p = 0.018], confirming the findings of the Cox regression analysis. In this setting, we again stratified according to the M-stage of patients. On the one hand, patients with metastatic disease seemed to benefit from having ePDT irrespective of the time point of ePDT (14.5 months *vs*. 18.8 months, p = 0.016). On the other hand, patients with locally advanced disease (M0 stage) benefited from having at least one eRFA in our cohort (12.7 months *vs*. 19.7 months, p = 0.042). We detected no difference on OS within the opposite scenario for both treatment groups (ePDT in M0 stage and eRFA in M1 stage) (**Figures 4A–D**).

We also compared patient’s OS depending on their tumor localization (irrespective of their further local therapy) and stratified them again for their first local ablative therapy. In particular, patients with an eCCA Bismuth III–IV seemed to also benefit in terms of OS through eRFA compared with that through ePDT [starting with eRFA: 19.7 months (95% CI, 12.2–27.2) *vs*. starting with ePDT: 12.7 months (95% CI, 12.0–13.4); p = 0.009], whereas there was no benefit for eRFA compared with that for ePDT in patients with eCCA Bismuth I–II and distal eCCA [starting with eRFA: 12.2 months (95% CI, 5.6–18.8) *vs*. starting with ePDT: 11.8 months (95% CI, 6.7–16.9); p = 0.516] (**Supplemental Datas 3A, B**). Furthermore, the beneficial effect of eRFA in Bismuth III–IV remains irrespective of ablative therapy sequence [eRFA: 17.3 months (95% CI, 9.8–24.8) *vs*. no eRFA: 12.7 (95% CI, 6.5–18.9)], whereas no effect was detectable for ePDT [ePDT: 13.5 months (95% CI, 11.3–15.7) *vs*. no ePDT: 14.8 months (95% CI, 11.9–17.7] (**Supplemental Datas 3C, D**).

### Tolerability

The frequencies of adverse events with CTCAE grading ≥3 are listed in **Table 4**. The most common adverse events were cholangitis (27 patients, 43.5%), anemia (26 patients, 41.9%), infection (26 patients, 41.9%), thrombopenia (21 patients, 33.9%), and neutropenia (14 patients, 22.6%). The only statistically significant difference was observed for cholangitis with a higher frequency within the eRFA + SC group (60.7%, p = 0.025). The ePDT-inherent phototoxicity of the skin occurred in about a fifth of patients receiving ePDT (SC + ePDT + eRFA, 23.1%; SC + ePDT, 18.2%) and in none of the eRFA-alone group as expected.

**Table 4:** Adverse events.

Parameters	SC + ePDT (N = 22)		SC + eRFA (N = 28)		SC + ePDT + eRFA (N = 13)		P-value
	N	%	N	%	N	%	
**Leucopenia**	4	19.0%	5	17.9%	3	23.1%	0.917
**Thrombopenia**	11	52.4%	8	28.6%	2	15.4%	0.106
**Anemia**	12	57.1%	11	39.3%	3	23.1%	0.231
**Neutropenia**	7	33.3%	5	17.9%	2	15.4%	0.434
**Other hematotoxic side effects**	0	0.0%	3	10.7%	0	0.0%	0.147
**ALT elevation**	7	33.3%	3	10.7%	3	23.1%	0.178
**AST elevation**	1	4.8%	0	0.0%	0	0.0%	0.389
**Ascites**	3	14.3%	3	10.7%	1	7.7%	0.889
**Hepatic dysfunction**	0	0.0%	2	7.1%	0	0.0%	0.285
**Anorexia**	2	9.5%	2	7.1%	1	7.7%	0.968
**Fatigue**	1	4.8%	2	7.1%	0	0.0%	0.626
**Nausea**	1	4.8%	6	21.4%	2	15.4%	0.236
**Vomit**	2	9.5%	5	17.9%	2	15.4%	0.664
**Creatinine elevation**	0	0.0%	2	7.1%	0	0.0%	0.296
**Chronic kidney failure**	2	9.5%	1	3.6%	0	0.0%	0.456
**Skin rash**	1	4.8%	1	3.6%	0	0.0%	0.766
**Diarrhea**	0	0.0%	2	7.1%	1	7.7%	0.415
**Edema**	0	0.0%	0	0.0%	1	7.7%	0.120
**Mucositis**	1	4.8%	0	0.0%	1	7.7%	0.357
**Allergic reaction**	1	4.8%	1	3.6%	0	0.0%	0.766
**Infection without neutropenia**	11	52.4%	12	42.9%	3	23.1%	0.366
**Infection with neutropenia**	0	0.0%	2	7.1%	0	0.0%	0.285
**Deep vein thrombosis**	1	4.8%	1	3.6%	0	0.0%	0.766
**Thromboembolic event**	6	28.6%	3	10.7%	0	0.0%	0.072
**Hyperbilirubinemia**	2	9.5%	2	7.1%	3	23.1%	0.256
**Jaundice**	1	4.8%	2	7.1%	0	0.0%	0.623
**Abdominal pain**	6	28.6%	10	35.7%	0	0.0%	0.052
**Cholangitis**	5	23.8%	17	60.7%	5	38.5%	**0.025**
**Biliary sepsis**	5	23.8%	2	7.1%	3	23.1%	0.215
**Infection (other than cholangitis)**	6	28.6%	3	10.7%	1	7.7%	0.205
**Weight loss**	0	0.0%	1	3.6%	1	7.7%	0.425
**Pains**	6	28.6%	7	25.0%	2	15.4%	0.772
**Pleural effusion**	1	4.8%	1	3.6%	0	0.0%	0.766
**Hypertension**	1	4.8%	0	0.0%	1	7.7%	0.369
**Fever**	6	28.6%	4	14.3%	3	23.1%	0.537
**Ileus**	1	4.8%	0	0.0%	2	15.4%	0.084
**Phototoxic reaction**	4	18.2%			3	23.1%	1.000

Statistically significant P-Values are displayed in bold values.

## Discussion

Cholangiocarcinoma remains a malignancy with a poor prognosis, with the majority of patients being diagnosed in an advanced and nonresectable stage. Consequently, research to improve current first-line treatment options is crucial. In this study, we highlighted the beneficial efficacy of additional eRFA and/or ePDT to standard SC on OS. Both eRFA and ePDT provided similar survival benefits, but patients receiving SC and ePDT as well as eRFA showed a trend to better survival, suggesting a possible synergism between the two local therapies. In further subgroup analysis, a higher beneficial effect from eRFA was observed. Combined therapy was well tolerated, with cholangitis occurring more often in the eRFA group. Thus, endobiliary should be discussed as an additional local treatment in patients suffering from advanced eCCA.

Because SC is the guideline-conformed therapy standard, concomitant endobiliary therapies should be evaluated in light of this setting. Because of the comparatively new endobiliary techniques, the majority of studies primarily focused only on feasibility and procedural efficacy of ePDT and eRFA and, subsequently, on the oncologic outcomes (OS, PFS, and therapy response). Data on endobiliary ablative therapies in cohorts of patients with 100% SC treatment rate are scarce for eCCA, studies reporting on the comparison of ePDT and eRFA even more rare so. In this study, it was our aim to compare the impact of additional ePDT and/or eRFA in a cohort of patients with advanced eCCA receiving SC as first-line therapy.

Overall, the combination of both endobiliary therapies with first-line SC resulted in a median OS of 20.2 months, whereas the addition of ePDT resulted in 12.7 months and the addition of eRFA resulted in 13.8 months. However, the median OS of the whole cohort was 14.0 months, and the differences in OS were not statistically significant (p = 0.112).

The ABC-02 trial demonstrated a median OS of 11.7 months for first-line treatment with gemcitabine and cisplatin, and the recent TOPAZ-1 study displayed an OS of 12.8 months for the addition of durvalumab (11, 13). Restrictively, it should be stated, that these studies included intrahepatic CCA, gallbladder carcinomas, and eCCA, affecting survival and response data.

Retrospective trials on real world cohorts including patients with exclusively eCCA display an OS of 16.3–20.0 months for SC and concomitant ePDT and 16.0–17.3 months for SC and concomitant eRFA (24, 30, 32–34, 38, 39).

Because of the low incidence of CCA, most of the studies are based on small cohorts. For larger cohorts, only reviews and meta-analyses provide data on OS of patients with CCA and, as already mentioned, accepting a higher heterogeneity in baseline and therapy characteristics. A recently published review and meta-analysis reported an OS of 11.9 months for patients treated with ePDT and 12 months for treatment with eRFA. In addition, the subgroup analysis showed a pooled median OS of 13.6 months for SC + ePDT confirming our OS for this group (40). However, the heterogeneity makes comparison with the results of the present study susceptible to bias. Only one trial published survival data on a direct intra-center comparison between ePDT and eRFA but without evaluation of possible synergistic effects of SC: Strand et al. reported an OS of 9.6 months for eRFA and 7.5 months for ePDT without a statistically significant difference (p = 0.799) (37).

Regarding PFS and response rates, the pivotal trials ABC-02 and TOPAZ-1 reported a median PFS of 8 months and 7.2 months for the gemcitabine + cisplatin group and the durvalumab + gemcitabine + cisplatin group, respectively. Furthermore, the ORR was 19.0% (ABC-02) and 26.7% (TOPAZ-1) and DCR was 79.0% and 85.3%, respectively (11, 13). A recent study from Japan retrospectively analyzing the effect of eRFA in combination with gemcitabine and cisplatin reported a PFS of 8.6 months, which is slightly shorter than the results of a previous publication from our group that showed PFS of 12.9 months for the combination group (34, 39). Inoue et al. also reported DCR of 82.0% and ORR of 18.0% (39). Reliable data on PFS or therapy response for SC + ePDT are not available to date. Compared with the published literature, the data of the present study seem to be in line with a median PFS of 6.4 months (SC + ePDT), 8.0 months (SC + eRFA), and 6.7 months (SC + ePDT + eRFA). Of our study groups, the SC + eRFA group had the best ORR of 21.4% compared with the SC + ePDT group of 4.5% and the SC + ePDT + eRFA group of 7.7%, whereas DCR was quite similar for the SC + eRFA group (75.0%) and the SC + ePDT + eRFA group (76.9%) and slightly less for the SC + ePDT group (54.5%).

The question as to which therapy fits best for which patient remains of high interest in palliation of eCCA. We tried to approach the question with a multivariate and subgroup analysis. Our data identified several interesting constellations. On the one hand, in the case of metastatic disease, patients treated with ePDT, irrespective of treatment point, seemed to benefit statistically significant in terms of OS. On the other hand, in the case of non-metastatic disease, the application of eRFA demonstrated improved survival in comparison to non-eRFA patients. Furthermore, the beneficial effect of eRFA over ePDT was especially seen in patients with Bismuth III–IV eCCA but not in Bismuth I–II eCCA. In line with the subgroup analysis, the multivariate analysis identified eRFA as an independent predictor of prolonged survival, together with use of metal stents.

However, these data need to be interpreted with caution, although the results match former publications. The positive influence of more than one line of SC on OS in CCA has been demonstrated in former studies concentrating on chemotherapy and, according to our data, seems to be confirmed in cohorts with eCCA and concomitant endobiliary ablative therapies (15). Importantly, a possible lead-time bias, especially inside the triple-therapy group, should be kept in mind as a possible confounding factor. Furthermore, the significant survival benefit of eRFA for Bismuth III–IV patients was also described by Bokemeyer et al. who reported an OS of 11.2 months for eRFA *vs*. 7.3 months for non-eRFA (p = 0.046) in this localization group, but with a SC rate of 31% within the cohort. The advantageous effect of eRFA for non-metastatic eCCA is in agreement with a retrospective propensity score-matched analysis from China, which presented an OS of 11.5 *vs*. 7.4 months (p < 0.001) for M0 *vs*. M1 (41).

Survival data for the application of SC and both endobiliary techniques during the first-line treatment, a “triple therapy” (SC + ePDT + eRFA), have not been published before. Only one trial focusing on ePDT highlighted the positive prognostic effect of eRFA within their regression analysis (32). With a median OS of 20.2 months, this treatment group outperforms the other groups using only ePDT or only eRFA as additional endobiliary therapy, but the survival difference lacks statistical significance. Nevertheless, it must to be highlighted that the baseline characteristics onset of any cancer-specific therapy were similar between the three treatment groups. However, the triple-therapy group received significantly more often a second-line SC and that could positively affect the OS (14, 42, 43). Potentially, the trend to a longer OS within the triple group might be an indication for summation potential of the beneficial effects of concomitant ePDT and eRFA. In this context, the prospective trial by Albers et al. is of high interest. They randomized patients with malignant biliary obstruction to self-expandable metal stent (SEMS) only (n = 44) or eRFA followed by SEMS insertion (n = 42) and reported no difference in either stent patency or OS. Elementary differences compared with those in our cohort of patients were the frequency of SC within the SEMS + eRFA group (40%) and the number of eRFA sessions (44). The prospective trial was designed to perform one eRFA followed by SEMS implantation, whereas our patients received repeated eRFA and ePDT, respectively. Therefore, the repeated endobiliary ablative therapy in combination might be a crucial aspect in superior survival of these patients. Nevertheless, the prospective data are of high value and might be extended in light of repeated eRFA.

Our safety and tolerability analysis revealed almost similar adverse event rates for all three treatment groups. The only exceptions were the higher frequency of cholangitis in the SC + eRFA group and the expected higher phototoxicity that only occurred in the ePDT-receiving groups. Our data concerning comparison of cholangitis frequency in ePDT *vs*. eRFA are in line with the study of Strand et al. who also reported a higher frequency for eRFA treatment. The authors supposed that the differences in further endoscopic treatment between the eRFA and ePDT arm might be the reason for the different cholangitis rates (37). Further publications reported the cholangitis incidence vice versa. Schmidt et al. as well as a large meta-analysis demonstrated higher cholangitis frequencies for the ePDT group (30%/23.4%) compared with that for the eRFA group (14%/9.5%) (40, 45). Interestingly, our SC + ePDT + eRFA group had a lower cholangitis frequency than the SC + eRFA group despite a comparatively longer OS and, therefore, more time to develop an episode of cholangitis. Patients receiving ePDT had a pooled phototoxicity rate of 20% in our cohort, which is somewhat higher than described in the meta-analysis of Mohan et al. but still within an acceptable range (40).

Several limitations should be kept in mind when interpreting the results of the present study. First, because of the retrospective study design, a possible selection bias cannot be excluded. Second, the immortal-time bias might affect the OS in our cohort, especially concerning second-line SC. Third, the limited number of patients might affect the statistical power of the study. Hence, the subgroup analysis in particular needs to be interpreted with caution. Fourth, the expertise of a tertiary center might affect the patient’s outcome, and the generalizability of these results needs to be confirmed outside from a specialized center. However, in comparison with the published literature and bearing the relatively low incidence of eCCA in mind, a total of 63 included patients can be respectable. Last, the direct comparison to a chemotherapy-alone group would add valuable insights to the effect of endobiliary techniques. Because our chemotherapy-alone group differed significantly in their tumor stage, we did not manage to form a robust group for comparison without adding a relevant selection bias.

In summary, the present study provides first evidence on survival and therapy response for eCCA under SC with concomitant combined endobiliary therapy (ePDT and eRFA). Furthermore, to the best of our knowledge, we performed the first comparative analysis for SC + ePDT *vs*. SC + eRFA in first-line treatment of eCCA. Both endobiliary therapies seem to be beneficial in terms of survival without increased toxicity. Thus, endobiliary PDT and/or RFA should be discussed as additional local therapies before starting SC.

Because the approval of durvalumab as addition to the long-standing SC standard with gemcitabine and cisplatin in 2022 as a result of the TOPAZ-1 trial, future studies should focus on the analysis of endobiliary ablative therapies in combination with immunochemotherapies.

## Data availability statement

The raw data supporting the conclusions of this article will be made available by the authors, without undue reservation.

## Ethics statement

The studies involving human participants were reviewed and approved by Ethics committee of the Medical Faculty of the University Bonn. The patients/participants provided their written informed consent to participate in this study.

## Author contributions

CM: acquisition of data, analysis and interpretation of data, drafting of the manuscript, and study concept and design. OK: acquisition of data, analysis and interpretation of data, drafting of the manuscript, and study concept and design. TZ: clinical procedures and critical revision of the manuscript for important intellectual content. FS: critical revision of the manuscript for important intellectual content. RM: clinical procedures and critical revision of the manuscript for important intellectual content. DK: clinical procedures and critical revision of the manuscript for important intellectual content. LD: clinical procedures and critical revision of the manuscript for important intellectual content. MT: critical revision of the manuscript for important intellectual content. MM: critical revision of the manuscript for important intellectual content. TG: critical revision of the manuscript for important intellectual content. HM: critical revision of the manuscript for important intellectual content. SM: critical revision of the manuscript for important intellectual content. JK: critical revision of the manuscript for important intellectual content. CS: critical revision of the manuscript for important intellectual content. TW: clinical procedures, acquisition of data, analysis and interpretation of data, drafting of the manuscript, and study concept and design. MG-C: clinical procedures, acquisition of data, analysis and interpretation of data, drafting of the manuscript, and study concept and design.
