# Supplementary figures and images for: Comparison between regular additional endobiliary radiofrequency ablation and photodynamic therapy in patients with advanced extrahepatic cholangiocarcinoma under systemic chemotherapy
Source: Front Oncol. 2023 Aug 29;13:1227036. doi: 10.3389/fonc.2023.1227036 (PMC10497756; doi:10.3389/fonc.2023.1227036)

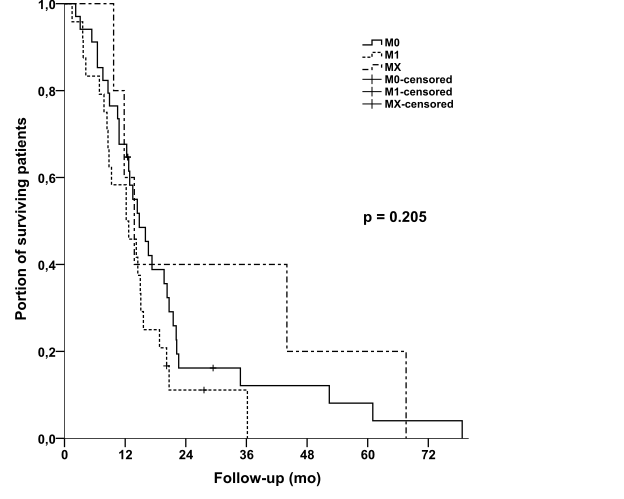

Supplement: Supplementary file 1 [file Image_1.tiff]

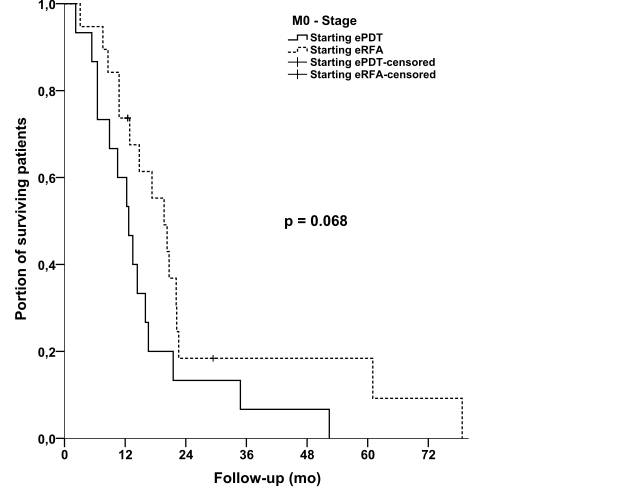

Supplement: Supplementary file 2 [file Image_2.tiff]

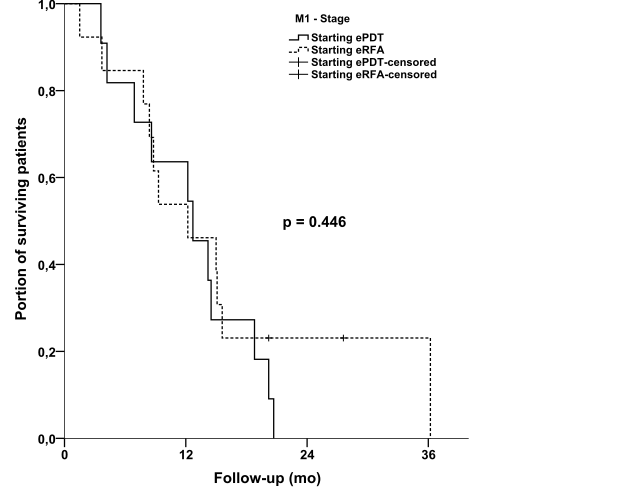

Supplement: Supplementary file 3 [file Image_3.tiff]

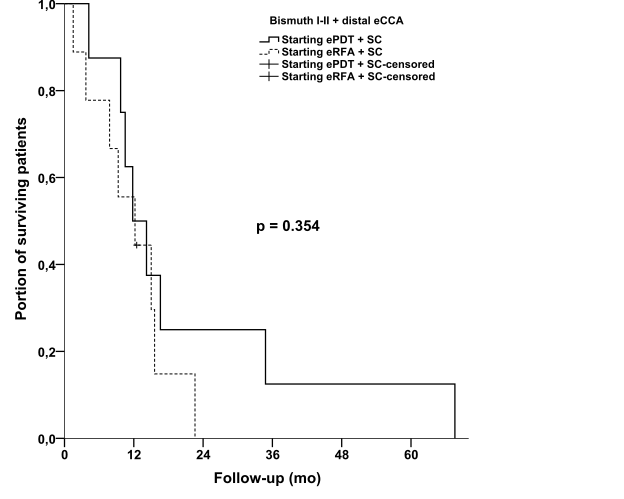

Supplement: Supplementary file 4 [file Image_4.tiff]

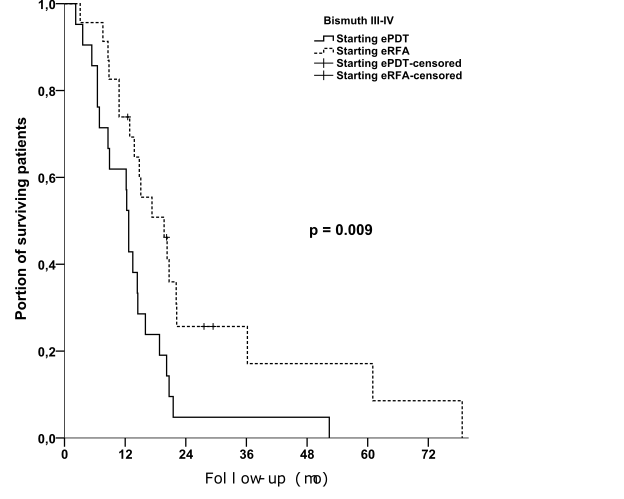

Supplement: Supplementary file 5 [file Image_5.tiff]

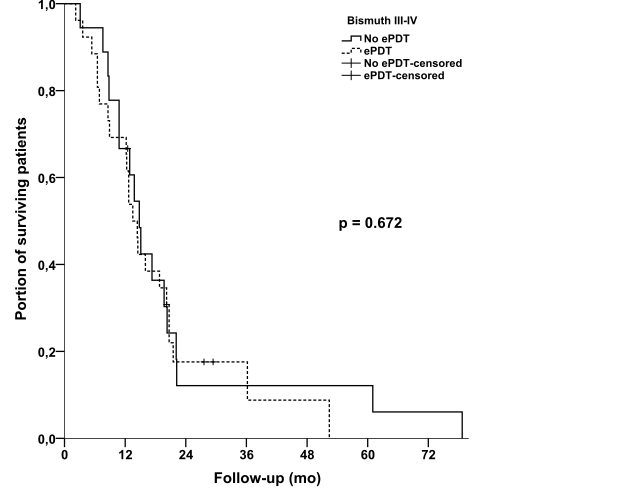

Supplement: Supplementary file 6 [file Image_6.tiff]

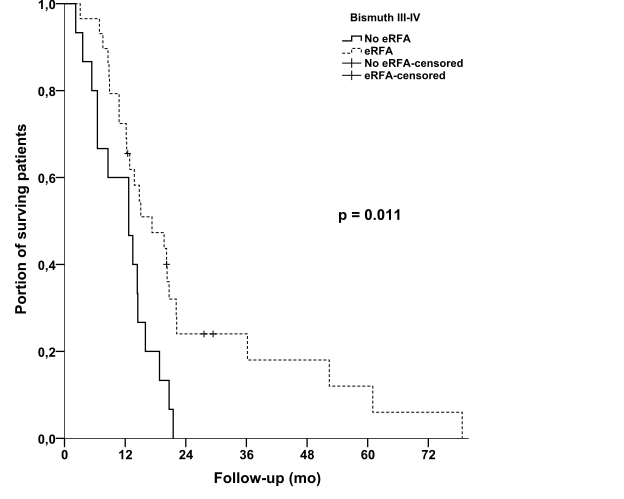

Supplement: Supplementary file 7 [file Image_7.tiff]
